# Biomarkers associated with low, moderate, and high vastus lateralis muscle hypertrophy following 12 weeks of resistance training

**DOI:** 10.1371/journal.pone.0195203

**Published:** 2018-04-05

**Authors:** Christopher B. Mobley, Cody T. Haun, Paul A. Roberson, Petey W. Mumford, Wesley C. Kephart, Matthew A. Romero, Shelby C. Osburn, Christopher G. Vann, Kaelin C. Young, Darren T. Beck, Jeffrey S. Martin, Christopher M. Lockwood, Michael D. Roberts

**Affiliations:** 1 School of Kinesiology, Auburn University, Auburn, AL, United States of America; 2 Department of Cell Biology and Physiology, Edward Via College of Osteopathic Medicine–Auburn Campus, Auburn, AL, United States of America; 3 Lockwood LLC, Draper, UT, United States of America; University of Birmingham, UNITED KINGDOM

## Abstract

We sought to identify biomarkers which delineated individual hypertrophic responses to resistance training. Untrained, college-aged males engaged in full-body resistance training (3 d/wk) for 12 weeks. Body composition via dual x-ray absorptiometry (DXA), vastus lateralis (VL) thickness via ultrasound, blood, VL muscle biopsies, and three-repetition maximum (3-RM) squat strength were obtained prior to (PRE) and following (POST) 12 weeks of training. K-means cluster analysis based on VL thickness changes identified LOW [n = 17; change (mean±SD) = +0.11±0.14 cm], modest (MOD; n = 29, +0.40±0.06 cm), and high (HI; n = 21, +0.69±0.14 cm) responders. Biomarkers related to histology, ribosome biogenesis, proteolysis, inflammation, and androgen signaling were analyzed between clusters. There were main effects of time (POST>PRE, p<0.05) but no cluster×time interactions for increases in DXA lean body mass, type I and II muscle fiber cross sectional area and myonuclear number, satellite cell number, and macronutrients consumed. Interestingly, PRE VL thickness was ~12% greater in LOW versus HI (p = 0.021), despite POST values being ~12% greater in HI versus LOW (p = 0.006). However there was only a weak correlation between PRE VL thickness scores and change in VL thickness (r^2^ = 0.114, p = 0.005). Forced post hoc analysis indicated that muscle total RNA levels (i.e., ribosome density) did not significantly increase in the LOW cluster (351±70 ng/mg to 380±62, p = 0.253), but increased in the MOD (369±115 to 429±92, p = 0.009) and HI clusters (356±77 to 470±134, p<0.001; POST HI>POST LOW, p = 0.013). Nonetheless, there was only a weak association between change in muscle total RNA and VL thickness (r^2^ = 0.079, p = 0.026). IL-1β mRNA levels decreased in the MOD and HI clusters following training (p<0.05), although associations between this marker and VL thickness changes were not significant (r^2^ = 0.0002, p = 0.919). In conclusion, individuals with lower pre-training VL thickness values and greater increases muscle total RNA levels following 12 weeks of resistance training experienced greater VL muscle growth, although these biomarkers individually explained only ~8–11% of the variance in hypertrophy.

## Introduction

Resistance training is a potent stimulus for skeletal muscle fiber hypertrophy. Well-known mechanisms associated with this adaptive response include repetitive post-bout increases in muscle protein synthesis (MPS) [1] as well as increases in satellite cell proliferation and myonuclear accretion [2]. Recent data [3, 4] and commentaries [5, 6] have also suggested ribosome biogenesis is critical for muscle hypertrophy given that ribosomes catalyze MPS. Ribosome biogenesis involves the coordinated action of transcription factors and transcriptional co-activators [e.g., v-Myc Avian Myelocytomatosis Viral Oncogene Homolog (c-Myc), Upstream Binding Factor (UBF), and others] recruiting RNA polymerase I (Pol I) to repetitive rDNA promoter regions to facilitate 47S pre-rRNA transcription [6, 7]. Moreover, rDNA transcription is seemingly rate-limiting in the process of ribosome biogenesis [8]. There is evidence to suggest that myofiber growth is abrogated with Pol I inhibition in vitro [4], which underscores the importance of Pol I activity in facilitating muscle growth. The process of ribosome biogenesis is also highly intricate in that it involves chromatin remodeling via complexes containing proteins such as Williams-Beuren Syndrome Chromosomal Region 10 Protein (WSTF) and SWI/SNF family member proteins [9]. However, aside from the abovementioned studies, there is limited evidence examining if various markers of ribosome biogenesis coincide with skeletal muscle hypertrophy following resistance training in humans.

Past studies from Bamman’s group have used K-means cluster analysis to delineate molecular characteristics between low-/non-, moderate- and high hypertrophic responders to resistance training [4, 10, 11]. Notably, this statistical approach has been extensively used over the past 50 years and possesses great utility given that it implements a systematic and unbiased algorithm to classify response clusters based on a criterion variable [12]. Using this approach, Petrella et al. [10] reported that resistance training-induced increases in satellite cell counts were greater in individuals experiencing an “extreme” muscle fiber cross sectional area (fCSA) increases to resistance training (termed XTR responders) compared to individuals experiencing a minimal hypertrophic response to resistance training (termed NON responders). Follow-up analyses indicated XTR responders experienced robust increases in the mRNA expression of genes related to growth factor signaling and satellite cell activity following 16 weeks of resistance training (e.g., different spliced variants of Insulin-Like Growth Factor-1 and Myogenin) [13]. Furthermore, a transcriptome-wide interrogation of these same subjects revealed that mRNAs related to ribosome biogenesis were up-regulated whereas mRNAs related to inflammation were down-regulated in XTR versus NON responders [11]. Given that heightened inflammation can increase muscle proteolysis [14, 15], the inability of low or non-hypertrophic responders to downregulate inflammation during resistance training may lead to a stagnation in muscle growth. Beyond these data from Bamman’s group, Mitchell et al. [16] reported that increases in skeletal muscle androgen receptor protein levels were correlated with myofiber hypertrophy following 12 weeks of resistance training in humans.

We recently published an investigation in untrained, college-aged males which tested the potential anabolic effects of L-leucine or protein supplementation over 12 weeks of resistance training [17]. Herein, we adopted the K-means cluster approach similar to Bamman’s laboratory [10], but instead of clustering groups based on changes in mean fCSA we generated three clusters based upon changes in vastus lateralis (VL) thickness assessed via ultrasound and identified low-responders (LOW), modest responders (MOD), and high responders (HI). Notably, VL ultrasound thickness was used as our criterion variable for muscle hypertrophy given that tracking muscle thickness changes via ultrasound may be more sensitive than dual x-ray absorptiometry (DXA) for tracking lean body mass changes [18]. Further, while using changes in fCSA as a clustering variable was deliberated, Lexell’s classical work suggests the number of muscle fibers within the VL can appreciably differ on an individual basis in younger men (calculated 95% confidence interval = 433,191 to 522,809 fibers) [19]. As a conceptual example, if two individuals experience similar increases in VL thickness following resistance training, we posit that the individual with more muscle fibers within the VL likely does not experience greater absolute increases in fCSA relative to the individual with less fibers despite the fact that the VL muscle hypertrophied to a similar degree. Following our VL thickness clustering, we sought to examine if pre-training levels or training-induced changes in body composition metrics along with total satellite cell counts, ribosome biogenesis markers, androgen signaling markers, or inflammatory and proteolytic markers differed between clusters. We hypothesized satellite cell counts, ribosome biogenesis markers, and/or androgen signaling markers would be greater at baseline or following training in HI responders versus other clusters, whereas these variables would be lower at baseline or less impacted by resistance training in LOW or MOD responders. Furthermore, we hypothesized inflammatory and proteolysis markers would be greater at baseline or following resistance training in the LOW responders relative to other cluster groups.

## Materials and methods

### Study protocol

Prior to initiating this study, the protocol was reviewed and approved by the Auburn University Institutional Review Board (IRB), and was in compliance with the Helsinki Declaration (approved protocol #: 15–320 MR 1508; IRB contact: irbadmin@auburn.edu). Participants provided written consent and completed a health history questionnaire to detect potential risk factors that might be aggravated by strenuous physical activity or skeletal muscle biopsies.

Untrained (i.e., at least 6 months of no structured resistance training), college-aged males (n = 67) from our previously published study [17] were stratified for analyses in the current study. Participants performed full-body resistance training sessions three days per week for 12 weeks. Each training session consisted of free-weighted exercises (i.e., barbell back squats, barbell bench press, barbell deadlifts, and barbell bent-over rows) and abdominal crunches. An undulating periodization model of resistance exercise shown to result in significant muscle hypertrophy and strength improvement in college-aged males was employed [20]. The first bout of training each week consisted of each barbell movement being performed for 4 sets of 10 repetitions, the second bout consisted of each movement being performed for 6 sets of 4 repetitions, and bout three consisted of each movement being performed for 5 sets of 6 repetitions. Loads lifted for each barbell movement were gradually increased on a per participant basis over the course of the study where ~50% of estimated one-repetition maximum (1-RM) was employed for each movement during week 1 of the study, with loads increasing to ~110% of initial estimated 1-RMs by the latter end of training. In the event that a load could not be executed with proficient technique for an exercise in a given training bout, weight was reduced on a per participant basis accordingly so that the next set could be executed. Training volumes for all participants were recorded throughout the entirety of the study.

Blood and muscle biopsy samples were taken from the antecubital vein and VL muscle, respectively, and these samples were obtained one week prior to training (PRE) and 72 hours following the last training bout (POST) around the same time of day (± 2 hours) at least 4 hours following a meal. During these testing sessions, VL thickness measures were taken via ultrasonography and lean body mass was assessed using dual x-ray absorptiometry (DXA). PRE and POST 3-RM back squat strength tests were also performed according to recommendations set forth by the National Strength and Conditioning Association [21]. Four-day food logs were completed by participants prior to the PRE and POST visits, and calorie and macronutrient intakes were analyzed using open-sourced software (https://www.myfitnesspal.com), which has been validated by past research [22] and has been used by others performing resistance training interventions [23, 24]. Readers are directed to Mobley et al. [17] for more in depth descriptions of PRE and POST testing batteries as well as the training protocol. Additionally, all methods related to body composition, serum and tissue analysis are in supporting information (S1 File 1. Analytical methods).

### Statistics

K-means cluster analysis (SPSS v 22.0; IBM Corp.; Armonk, NY, USA) based on changes in VL thickness following resistance training was used to identify three clusters similar to the methods of Stec et al. [4]. Following K-means clustering, Shapiro-Wilk testing of normality was conducted for all dependent variables. All variables for which significance was observed were square root-transformed for subsequent statistical testing (noted in the results). Given that the MOD cluster had more respondents relative to the low and high cluster, homogeneity of variance testing between clusters at PRE and POST was conducted on all dependent variables using Levene’s tests. Notably, all variables except for Pol I protein at POST had Levene’s test p-values >0.05. Thus, post hoc adjustments were not performed given that between-cluster variances were statistically similar in all but one variable. Select baseline dependent variables were analyzed between clusters using one-way ANOVAs with Tukey post hoc tests. Dependent variable comparisons over time were analyzed between clusters using 3×2 (cluster×time) mixed factorial repeated measures ANOVAs. If a significant cluster effect was observed, Tukey’s post hoc tests were performed to determine which clusters differed. If a significant cluster×time interaction was observed, PRE-to-POST dependent samples t-tests (corrected for multiple comparisons) were performed within each cluster, and one-way ANOVAs with Tukey post hoc tests at the PRE and POST time points were performed to determine between-cluster differences. Bivariate correlations were also performed on select variables which differed between clusters in order to better establish the degree of association that existed between biomarkers and change in VL thickness. All statistical analyses were performed using SPSS v22.0 (IBM Corp) and significance was established at p<0.05, although p-values “approaching significance” (i.e., 0.050<p<0.100) were also discussed given the exploratory nature of the investigation. All raw data can be found in supporting information (S2 File. Raw data).

## Results

### Cluster characteristics based upon VL thickness changes

**Fig 1:**
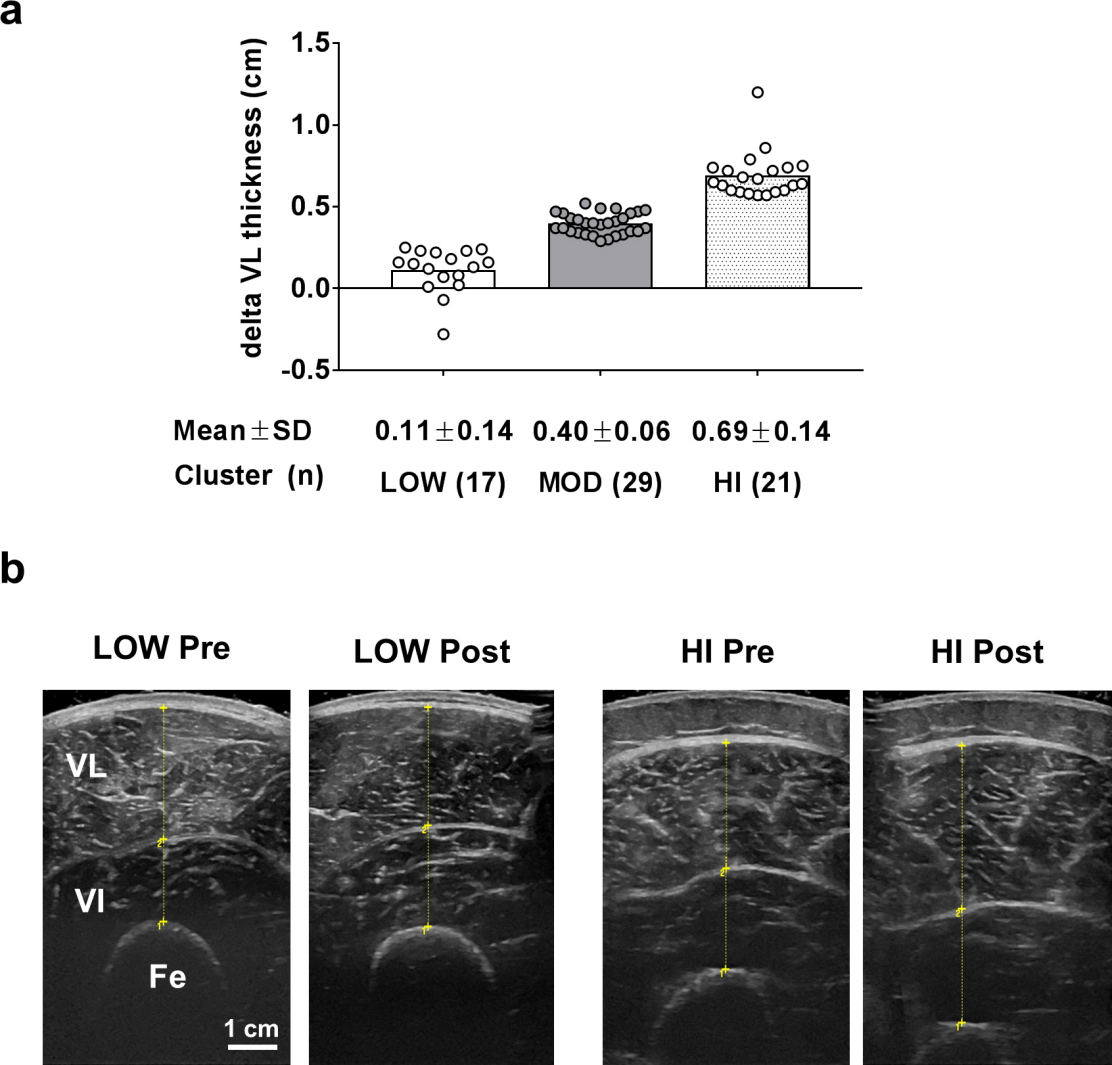
VL thickness as a clustering variable. Legend: K-means cluster analysis was used to differentiate LOW, MOD and HI responders (panel a). Data are presented as individual respondent values, bar graph values indicate group mean values, and mean ± standard deviation values are presented below each bar. Panel b depicts PRE versus POST representative ultrasound images (scale bar = 1 cm); abbreviations: VL, vastus lateralis; VI, vastus intermedius, Fe, femur.

For reference, Fig 1 illustrates the three clusters based upon changes in VL thickness following 12 weeks of resistance training. Cluster values for each group were as follows [means ± SD, (range)]: LOW = 0.11 ± 0.14 cm (-0.28 to 0.25 cm), MOD = 0.40 ± 0.06 cm (0.29 to 0.52 cm), HI 0.69 ± 0.14 cm (0.59 to 1.20 cm).

### Baseline characteristics and total training volume between clusters

Pre-training differences between clusters regarding age, body mass, and fiber type are presented in Table 1. Total logged training volume over the intervention is also presented in Table 1. Notably, there were no significant between-cluster differences in the baseline variables examined or total training volume lifted throughout the study (ANOVA p-value ranges = 0.239–0.405). As stated previously, participants consumed either maltodextrin placebo, L-leucine with maltodextrin (LEU) or different protein supplements [whey protein concentrate (WPC), hydrolyzed whey protein (WPH), soy protein concentrate (SPC), or maltodextrin placebo (PLA)] throughout the duration of this study [17]. The Chi-square p-value in Table 1 illustrates that the number of participants representing each level of supplement did not differ statistically. Therefore, the effect of supplement on the clustering of participants was likely not meaningful.

**Table 1:** Baseline characteristics and training volume between clusters.

Variable	LOW (n = 17)	MOD (n = 29)	HI (n = 21)	p-value
Age (years)	21±1	20±1	21±2	0.351
Body mass (kg)	79.0±9.1	76.7±11.3	74.5±8.7	0.405
Type II fiber (%)	67±10	63±11	68±10	0.239
Total volume (kg)	323,771±47241	323,471±47,148	305,484±44,856	0.341
**Number (%) of participants in supplement groups from Mobley et al. [17]**				
LEU	4 (24%)	5 (17%)	4 (19%)	**p-value**
WPC	3 (18%)	5 (17%)	7 (33%)	
WPH	3 (18%)	9 (31%)	0 (0%)	0.219
SPC	5 (29%)	5 (17%)	4 (19%)	
PLA	2 (12%)	5 (17%)	6 (29%)	

All data are presented as mean ± standard deviation values. Abbreviations: LEU, participants that supplemented twice daily with 3 g of L-leucine and 43 g of maltodextrin; WPC, participants that supplemented twice daily with 26 g of whey protein concentrate; WPH, participants that supplemented twice daily with 25 g of hydrolyzed whey protein; SPC, participants that supplemented twice daily with 39 g of soy protein concentrate; PLA, participants that supplemented twice daily with 44 g of maltodextrin.

### Self-reported macronutrient intakes between clusters

PRE and POST macronutrient intake differences between clusters are presented in Table 2. There were no cluster effects or cluster×time interactions for daily calorie, protein, carbohydrate, or fat intakes. However, there were significant main effects of time for all of these variables (POST>PRE, p<0.001).

**Table 2:** Self-reported macronutrient intakes.

Variable	PRE	POST	Statistics
Energy intake (kcal/d)			
LOW	1722±426	2440±521	Cluster p = 0.327 Time p<0.001 (POST>PRE) C×T p = 0.758
MOD	2033±604	2595±703	
HI	1924±492	2569±493	
Protein intake (g/d)			
LOW	84±20	142±44	Cluster p = 0.848 Time p<0.001 (POST>PRE) C×T p = 0.617
MOD	90±27	137±40	
HI	86±24	133±36	
Carbohydrate intake (g/d)			
LOW	185±53	251±62	Cluster p = 0.140 Time p<0.001 (POST>PRE) C×T p = 0.537
MOD	230±80	283±86	
HI	217±70	296±105	
Fat intake (g/d)			
LOW	68±17	99±28	Cluster p = 0.792 Time p<0.001 (POST>PRE) C×T p = 0.616
MOD	77±23	100±43	
HI	78±21	95±45	

Significant main time effects were observed for calorie and macronutrient intakes in all clusters (p<0.001). All data are presented as mean ± standard deviation values.

### Pre- to post-training changes in VL thickness and DXA lean body mass

**Fig 2:**
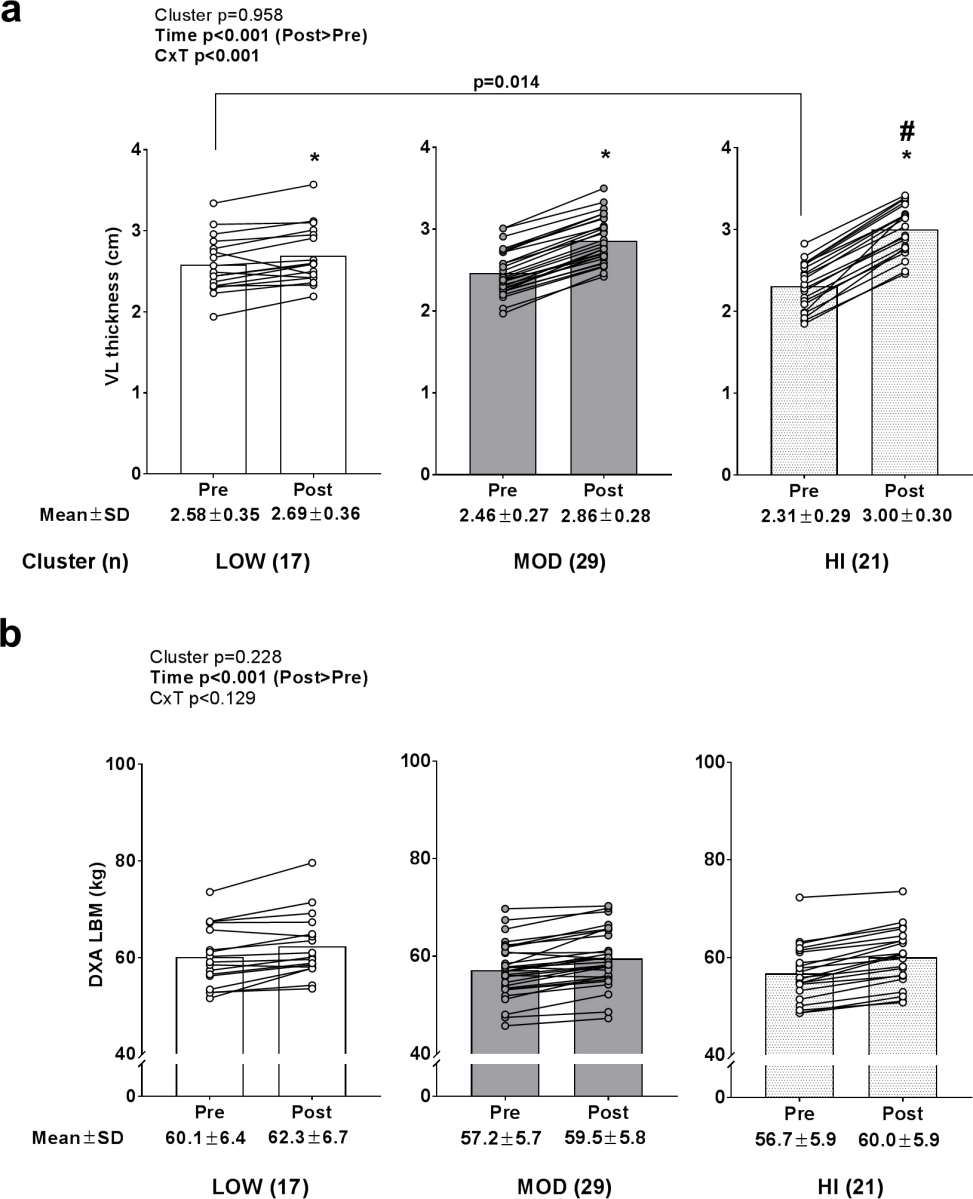
Pre and post-training VL thickness and DXA lean body mass values between clusters. Legend: All clusters presented with increases in VL thickness following resistance training (*, p<0.05) (panel a), although PRE levels were greater in LOW versus HI (p = 0.014) and POST levels were greater in HI versus LOW (#, p = 0.006). Panel b depicts PRE- to POST-training changes in DXA lean body mass (LBM), whereby only a time effect was observed (p<0.001). Data are presented as individual respondent values, bar graph values indicate group mean values, and mean ± standard deviation values are presented below each bar.

A significant time effect (POST>PRE, p<0.001) and cluster×time interaction (p<0.001) was observed for change in VL thickness (Fig 2A). All clusters experienced increases in VL thickness (p<0.05). PRE VL thickness values were greater in LOW versus HI (p = 0.014), whereas POST values were greater in HI versus LOW (p = 0.006). However there was only a weak correlation between baseline VL thickness scores and change in VL thickness values (r^2^ = 0.114, p = 0.005). A significant time effect (POST>PRE, p<0.001) was observed for DXA lean body mass changes (Fig 2B), but no cluster effect or cluster×time interaction existed.

### Changes in muscle fiber cross sectional area, myonuclear number, and satellite cell number between clusters

**Fig 3:**
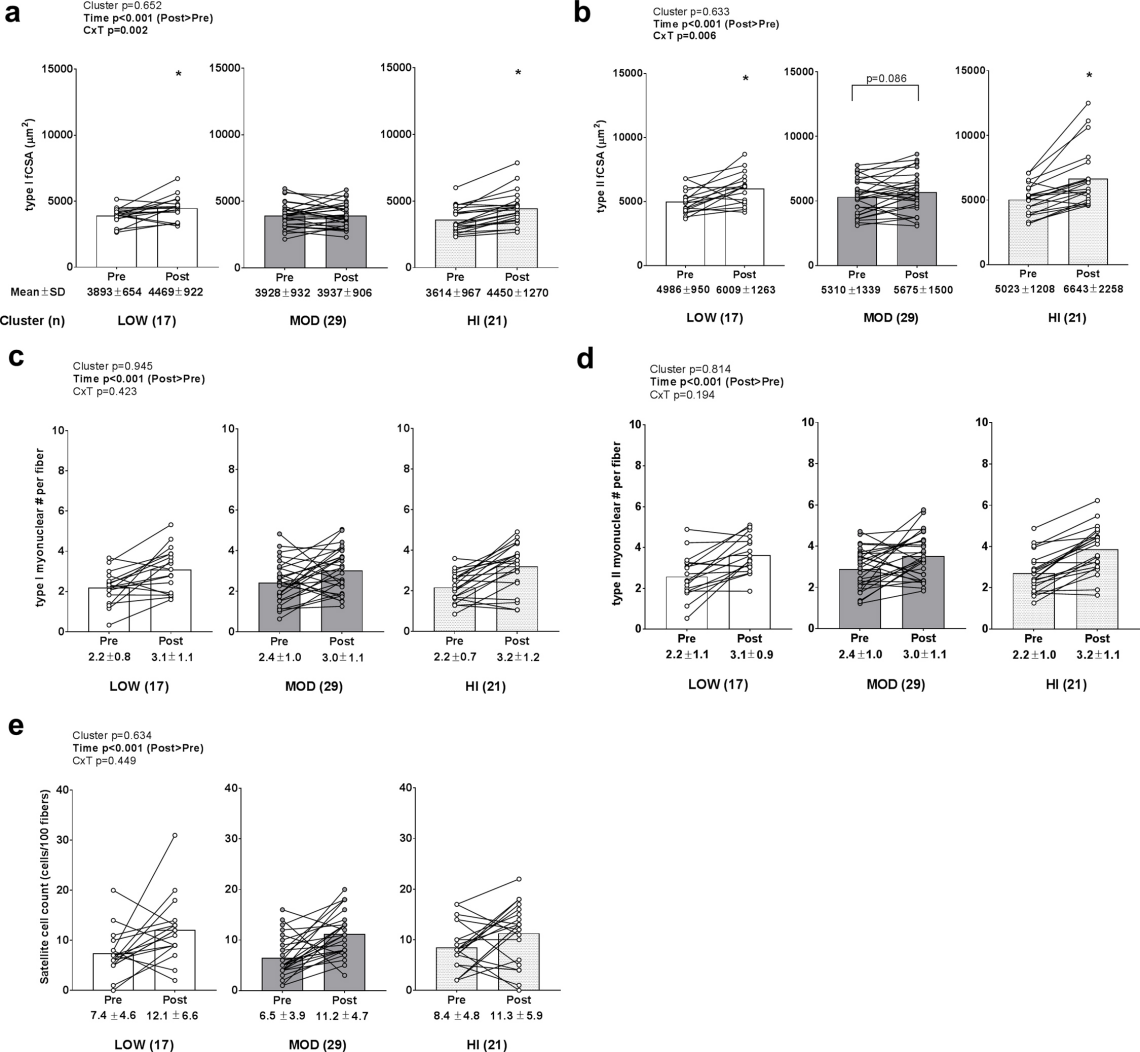
Change in muscle fiber cross sectional area, myonuclear number, and satellite cell number between clusters. Legend: Main effects of time existed for type I fCSA changes (POST>PRE, p<0.001) (panel a), type II fCSA changes (POST>PRE, p<0.001) (panel b), type I myonuclear number per fiber (POST>PRE, p<0.001) (panel c), type II myonuclear number per fiber (POST>PRE, p<0.001) (panel d), and satellite cell number (POST>PRE, p<0.001) (panel e). However, no cluster effects or cluster×time interactions existed. Data are presented as individual respondent values, bar graph values indicate group mean values, and mean ± standard deviation values are presented below each bar.

A significant time effect (POST>PRE, p<0.001) and cluster×time interaction (p = 0.002) were observed for change in type I fCSA (Fig 3A). LOW and HI clusters experienced increases in type I fCSA (p<0.05), although there were no PRE or POST differences in values between clusters. A significant time effect (POST>PRE, p<0.001) and cluster×time interaction (p = 0.006) was also observed for change in type II fCSA (Fig 3B). The LOW and HI clusters experienced increases in type II fCSA (p<0.05) and increases in the MOD cluster approached significance (p = 0.086). However, there were no PRE or POST differences in type II fCSA values between clusters. Only significant time effects (POST>PRE, p<0.001) were observed for change in type I fiber myonuclear number (Fig 3C), type II fiber myonuclear number (Fig 3D), and satellite cell number (data transformed prior to analysis due to non-normal distribution; Fig 3E).

### Changes in ribosome biogenesis markers between clusters

**Fig 4:**
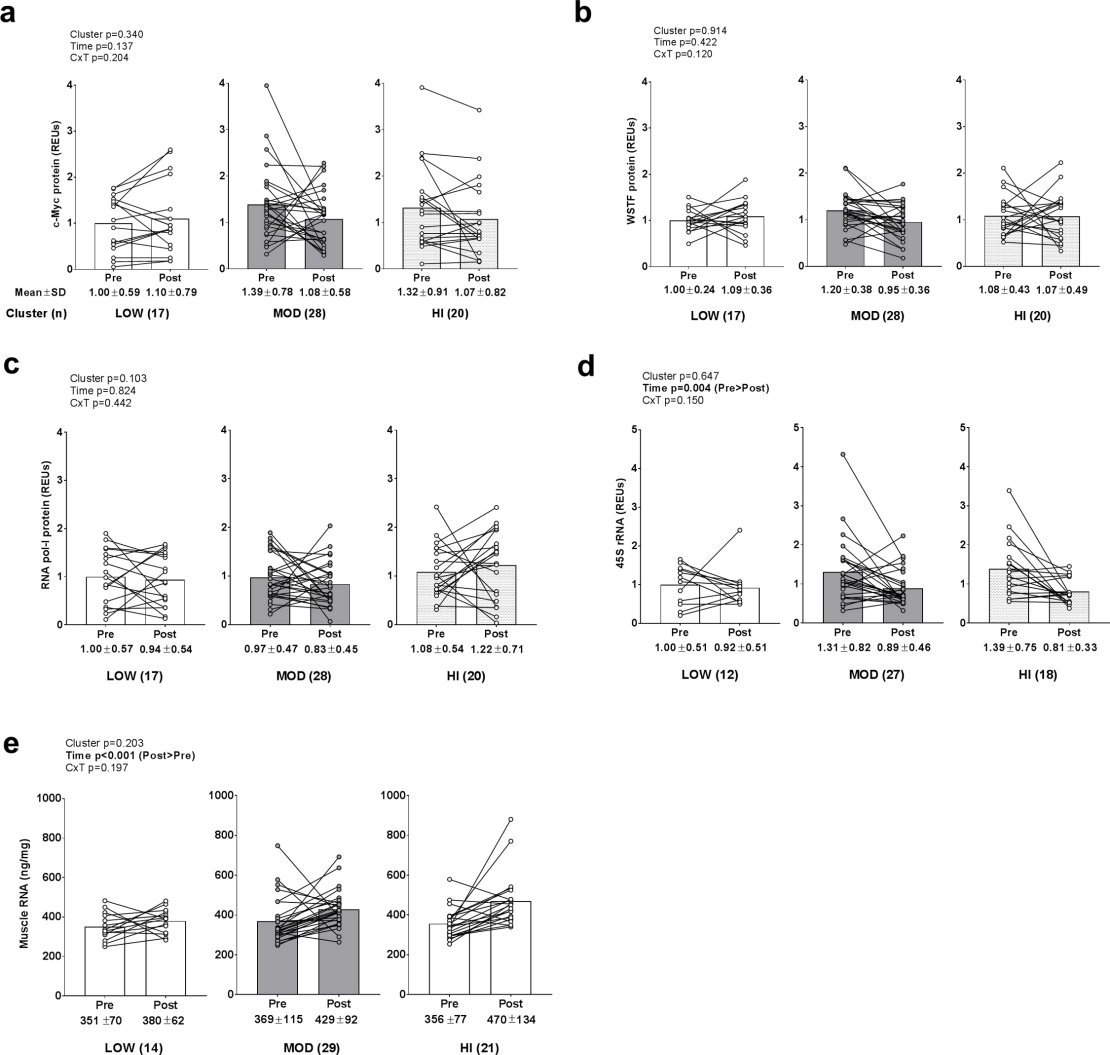
Alterations in ribosome biogenesis biomarkers between clusters. Legend: No main effects or cluster×time interactions existed for c-Myc protein (panel a), WSTF protein (panel b), or RNA pol-I protein levels (panel c). Main time effects existed for 45S rRNA levels (PRE>POST, p = 0.004) (panel d) and total RNA levels (POST>PRE, p<0.001) (panel e), but no main cluster effects or cluster×time interactions existed. Data are presented as individual respondent values, bar graph values indicate group mean values, and mean ± standard deviation values are presented below each bar.

No main effects or cluster×time interactions existed for c-Myc protein (data transformed prior to analysis due to non-normal distribution), WSTF protein, or RNA pol-I protein levels (Fig 4A–4C). Main time effects existed for 45S rRNA levels (data transformed prior to analysis due to non-normal distribution; PRE>POST, p = 0.002; Fig 4D) and total RNA levels (data transformed prior to analysis due to non-normal distribution; POST>PRE, p<0.001; Fig 4E), but no main cluster effects or cluster×time interactions existed. There was a weak but significant correlation between change in muscle total RNA and VL thickness (r^2^ = 0.079, p = 0.026).

### Androgen signaling biomarkers between clusters

**Fig 5:**
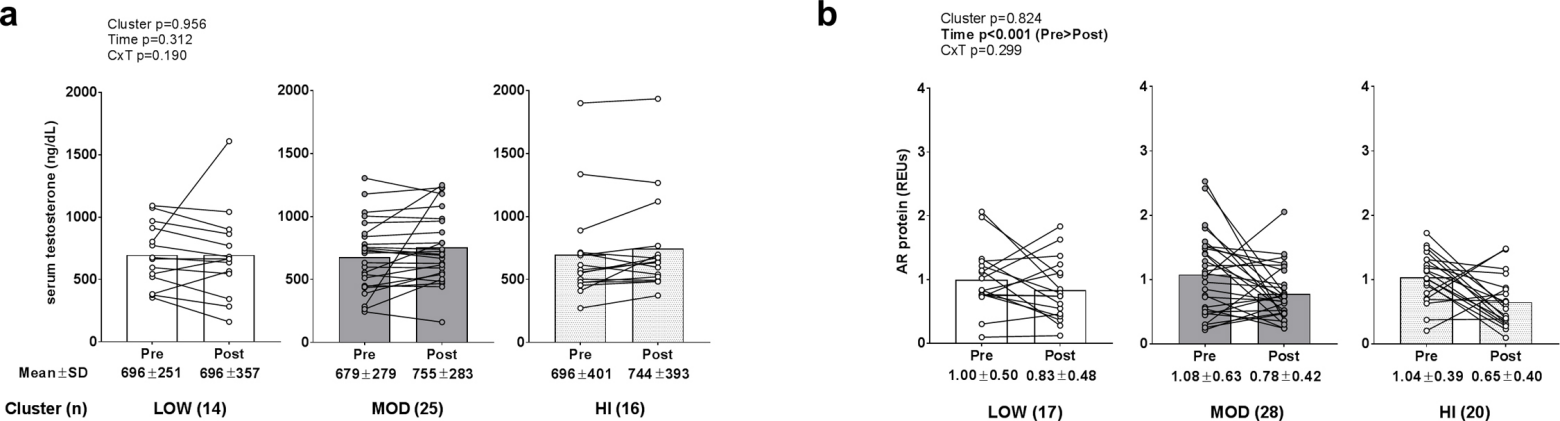
Alterations in androgen signaling biomarkers between clusters. Legend: No significant main effects or cluster×time interaction existed for serum total testosterone (panel a). A significant time effect existed for AR protein levels (PRE>POST, p<0.001) (panel b), but no main cluster effect or cluster×time interaction existed. Data are presented as individual respondent values, bar graph values indicate group mean values, and mean ± standard deviation values are presented below each bar.

No significant main effects or cluster×time interaction existed for serum total testosterone (data transformed prior to analysis due to non-normal distribution; Fig 5A). A significant time effect existed for AR protein levels (data transformed prior to analysis due to non-normal distribution; PRE>POST, p<0.001), but no main cluster effect or cluster×time interaction existed.

### Catabolic biomarkers between clusters

**Fig 6:**
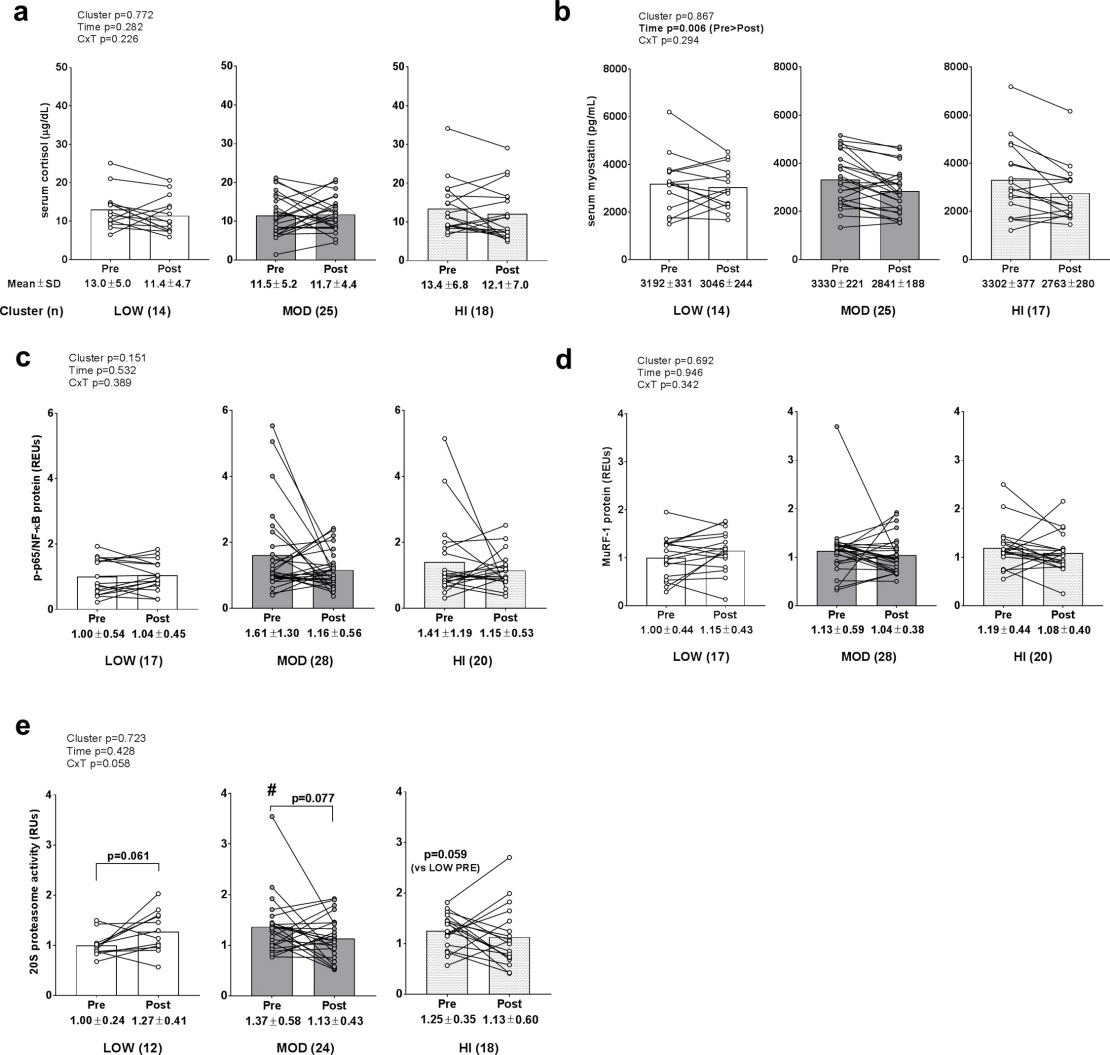
Alterations in catabolic biomarkers between clusters. Legend: No significant main effect or cluster×time interaction existed for serum cortisol (panel a). A significant time effect existed for serum myostatin levels (PRE>POST, p = 0.006), but no main cluster effect or cluster×time interaction existed (panel b). No significant main effects or cluster×time interactions existed for phosphorylated NF-κB protein levels (panel c), or MuRF-1 protein levels (panel d). A cluster×time interaction approached significance for 20S proteasome activity (p = 0.058) (panel e), and forced post hoc tests revealed: a) PRE levels were greater in MOD versus LOW (#; p = 0.020), b) higher POST versus PRE levels in the low group approached significance (p = 0.061), and c) higher PRE versus POST levels in the MOD group approached significance (p = 0.077). Data are presented as individual respondent values, bar graph values indicate group mean values, and mean ± standard deviation values are presented below each bar.

No significant main effect or cluster×time interaction existed for serum cortisol (Fig 6A). A significant time effect existed for serum myostatin levels (PRE>POST, p = 0.006) (Fig 6B), but no main cluster effect or cluster×time interaction existed. No significant main effects or cluster×time interactions existed for phosphorylated NF-κB protein levels (Fig 6C), or MuRF-1 protein levels (Fig 6D). Interestingly, a cluster×time interaction approached significance for 20S proteasome activity (p = 0.058; Fig 6E). Given that the interaction approached statistical significance, we performed forced post hoc tests which suggested that 20S proteasome activity was: a) at the PRE time point, greater in MOD versus LOW (p = 0.020) and trended higher in HI versus LOW (p = 0.068), b) trending higher at the POST time point compared to PRE within the LOW cluster (p = 0.061; threshold for significance is p<0.017 due to multiple t-tests), and c) trending lower at the POST time point compared to PRE within the MOD cluster (p = 0.077; threshold for significance is p<0.017 due to multiple t-tests). A weak negative association existed between change in 20S proteasome activity and change in VL thickness (r^2^ = 0.057, p = 0.083). Notably, all data included in Fig 6 were transformed prior to analysis due to these variables being non-normally distributed.

### Inflammatory and catabolic mRNA levels between clusters

**Fig 7:**
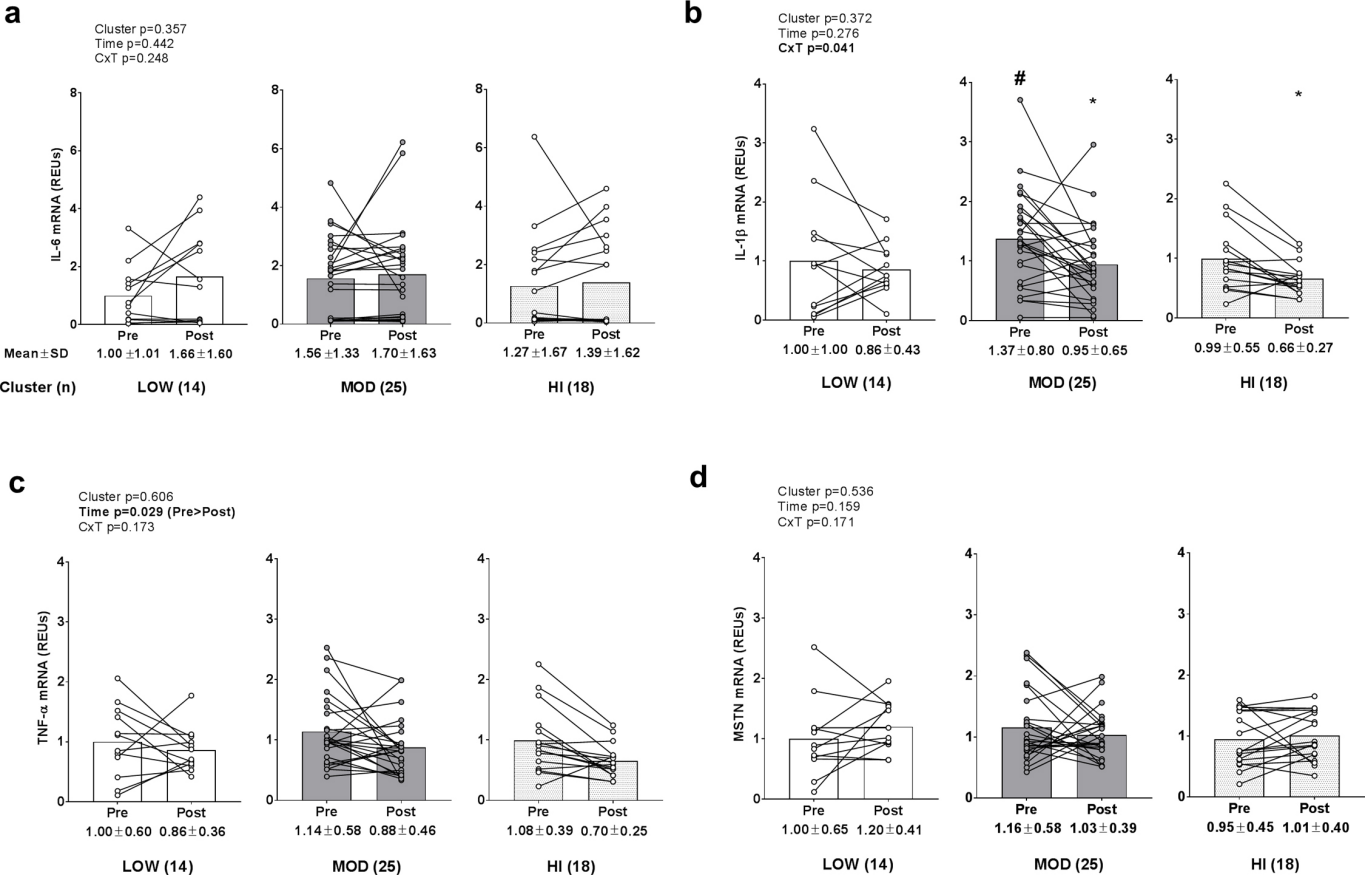
Alterations in inflammatory and catabolic mRNA levels between clusters. Legend: No significant main effects or cluster×time interaction existed for skeletal muscle IL-6 mRNA levels (panel a). A significant cluster×time interaction existed for existed for IL-1β mRNA (p = 0.029 (panel b), and post hoc tests revealed: a) PRE IL-1β mRNA levels were greater in MOD versus LOW (#; p = 0.050), and b) levels decreased in the MOD (*, p = 0.006) and LOW (*, p = 0.019) clusters following training. A significant time effect existed for TNF-α mRNA (PRE>POST, p = 0.029) (panel c), but no main cluster effect or cluster×time interaction existed. No significant main effect or cluster×time interaction existed for skeletal muscle MSTN mRNA levels (panel d). Data are presented as individual respondent values, bar graph values indicate group mean values, and mean ± standard deviation values are presented below each bar.

No significant main effects or cluster×time interaction existed for skeletal muscle IL-6 mRNA levels (Fig 7A). A significant cluster×time interaction existed for existed for IL-1β mRNA (p = 0.029; Fig 7B), but no main effects existed. Post hoc tests revealed: a) PRE IL-1β mRNA levels were greater in MOD versus LOW (p = 0.050), and b) levels decreased in the MOD cluster (p = 0.006; threshold for significance is p<0.017 due to multiple t-tests) and trended downward in the LOW (p = 0.019; threshold for significance is p<0.017 due to multiple t-tests) cluster following training. However, there was no significant association between VL thickness changes and IL-1β mRNA changes between clusters (r^2^ = 0.0002, p = 0.919). A significant time effect existed for TNF-α mRNA (PRE>POST, p = 0.029; Fig 7C), but no main cluster effect or cluster×time interaction existed. No significant main effect or cluster×time interaction existed for skeletal muscle MSTN mRNA levels (Fig 7D). Notably, all data in Fig 7 were transformed prior to analysis due to these variables being non-normally distributed.

### Lower-body strength changes between clusters

**Fig 8:**
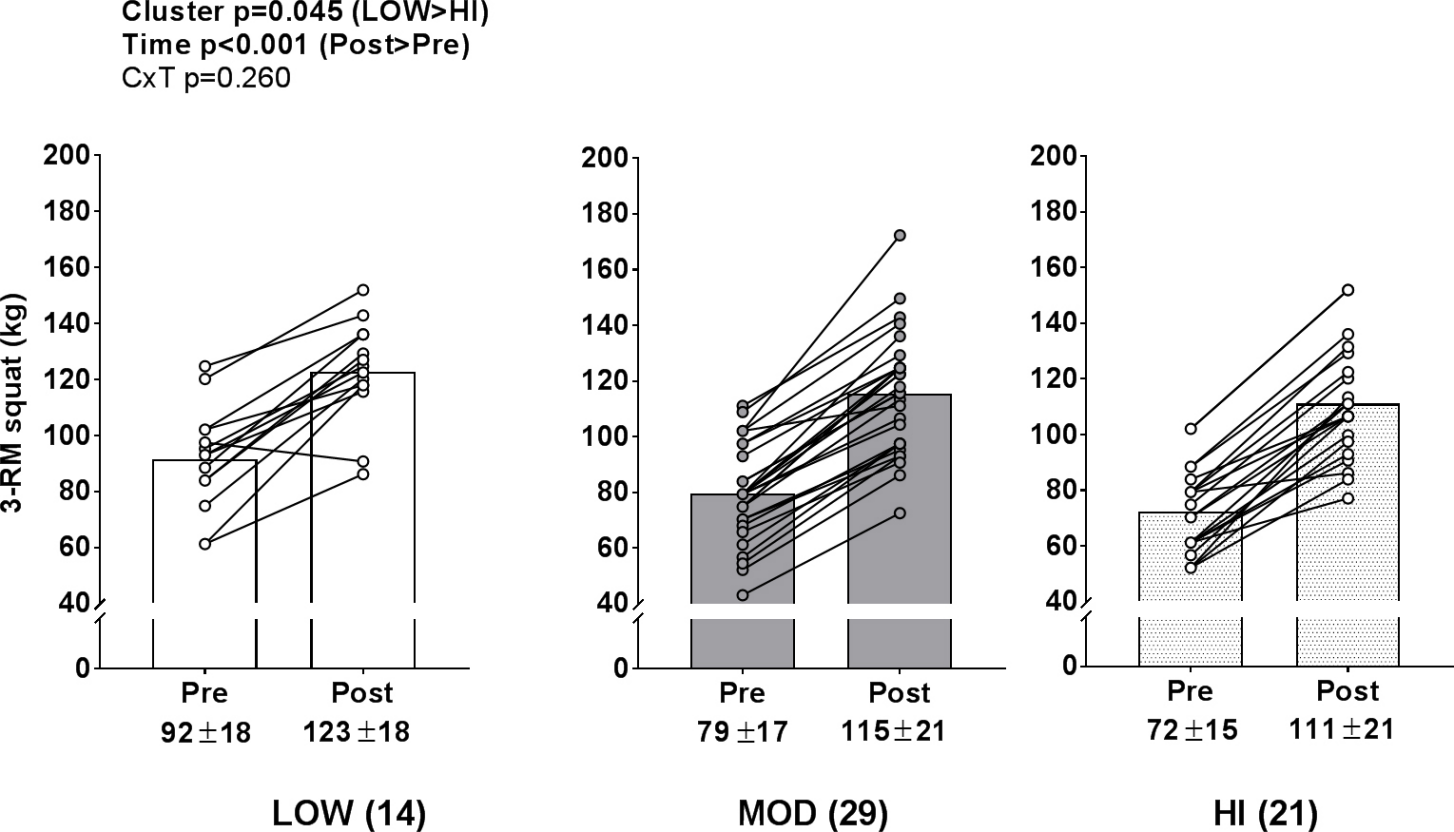
Lower body strength changes between clusters. Legend: A significant time effect (PRE>POST, p<0.001) and cluster effect (LOW>HI, p = 0.045) existed for 3-RM squat strength, but no cluster×time interaction existed. Data are presented as individual respondent values, bar graph values indicate group mean values, and mean ± standard deviation values are presented below each bar.

A significant time effect (PRE>POST, p<0.001) and cluster effect (LOW>HI, p = 0.045) existed for 3-RM squat strength (Fig 8B), but no cluster×time interaction existed.

## Discussion

The current study continues to expand upon past reports which have identified biomarkers delineating individual hypertrophic responses to resistance training. Herein, satellite cell counts increased in response to training, but no cluster×time interaction was observed. Our findings differ from data published by Bamman’s laboratory reporting heightened satellite cell counts following 16 weeks of resistance training in XTR versus other clusters [10]. It should be noted, however, that differences in study designs (i.e., participant population, training program, supplementation, and study duration) potentially lend to the discordant findings. Notably, younger and older males and females were included in Petrella et al. study, and an earlier publication by this group using many of these same participants reported that only younger males (not older males or females) experienced increases in satellite cell counts following 16 weeks of resistance training [25]. Collectively these data suggest that young, untrained males experience hypertrophy with concomitant increases in satellite cell number in response to resistance training. However, our data suggest that satellite cell increases in this population are not related to the degree of skeletal muscle hypertrophy as assessed via VL thickness.

Stec et al. [4] reported XTR responders presented rapid increases in type II hypertrophy accompanied by increases in ribosome density (i.e., total RNA per weight of assayed muscle) in response to only 4 weeks of resistance training. While we only observed a significant time effect for total RNA levels, we performed a forced post hoc analysis given that there was a high magnitude change score in the HI cluster (+32% following training) versus the LOW cluster (+8%). This forced post hoc analysis indicated the following (note that significance was not indicated on Fig 4E due to the lack of a significant interaction): a) muscle total RNA levels did not significantly increase in the LOW cluster (p = 0.253), but increased in the MOD (p = 0.009; threshold for significance is p<0.017 due to multiple t-tests) and HI clusters (p<0.001), and b) post training RNA levels were greater in the HI versus LOW cluster (p = 0.013). Thus, we posit that greater increases in ribosome biogenesis may delineate hypertrophic responses to resistance training as Stec et al. and others have reported in humans [3, 4]. However, our finding that change in muscle total RNA only explained ~8% in the variance in VL thickness changes (r^2^ = 0.079) implicates that this marker may not be a strong predictor of skeletal muscle hypertrophy.

In spite of ribosome density increasing with training, 45S pre-rRNA expression levels were down-regulated with resistance training regardless of cluster. Indeed, these findings differ from Stec et al. [4] and Figueiredo et al. [3] who reported that 4 and 8 weeks of resistance training, respectively, up-regulate 45S pre-rRNA levels. However, our findings suggest ribosome biogenesis likely operates via a negative feedback mechanism whereby the training-induced increase in ribosome density herein potentially promoted a downregulation of rDNA transcription. This hypothesis is not unfounded given that transcriptomic and MPS responses to resistance training operate in a similar fashion whereby a novel exercise stimulus elicits robust alterations in these variables relative to subsequent exercise bouts [26]. However, a single 45S primer set situated towards the 5’ end of the transcript was used as a proxy for ribosomal biogenesis which limits our ability to make definitive conclusions concerning how training affected expression levels.

Pro-inflammatory cytokines (e.g., TNF-α and IL-1β) up-regulate proteolytic activity in skeletal muscle [15, 27, 28]. Additionally, while IL-6 has several pleiotropic roles in skeletal muscle [29], rodent [14] and human data [30] suggest heightened IL-6 up-regulates skeletal muscle protein proteolysis. Muscle proteolysis is largely regulated by atrogene induction [31], and MuRF-1 is a muscle-specific E3 ligase that ubiquinates myofibrillar proteins (e.g., myosin heavy chains, troponin I, and other myosin-related proteins). Following E3-catalyzed poly-ubiquination, muscle proteins are degraded by the 26S proteasome which is made up of the 20S enzymatic core particle and 19S regulatory particle [32]. We observed a training effect regarding a down-regulation in TNF-α mRNA levels, and this is in agreement with past resistance training studies [33, 34]. Interestingly, IL-1β mRNA levels significantly decreased in the MOD cluster and trended downward in the HI cluster, but was not altered in the LOW cluster following training. Additionally, a cluster×time interaction for 20S proteasome activity approached significance (p = 0.058), and a forced *post-hoc* analysis suggested that activity trended upward in the LOW cluster following 12 weeks of resistance training. While speculative, these data could indicate that the inability to down-regulate select pro-inflammatory mRNAs and/or an induction in proteasome activity levels in LOW responders may have been partially responsible for the lower magnitude of hypertrophy in this group relative to other groups. However, the lack of modest or large associations between changes in IL-1β mRNA levels or 20S proteasome levels and changes in VL thickness suggest that these markers were minimally predictive of VL hypertrophy which limits the likelihood of the aforementioned hypothesis.

Testosterone is an anabolic sex hormone that binds as a ligand to androgen receptors and has been extensively studied regarding its ability to increase skeletal muscle mass through stimulating satellite cell proliferation [35, 36] and MPS [37, 38]. While others have reported increases in muscle AR protein content correlate with muscle hypertrophy [16], we observed that 12 weeks of resistance training downregulated AR content regardless of cluster. Hence, as with 45S rRNA, this observation likely suggests that repeated training bouts elicits a negative feedback loop regarding the AR protein expression. Additionally, there was no training effect or between-cluster effect or interaction regarding serum testosterone levels which seemingly agrees with other literature suggesting that changes in serum free or total testosterone with resistance training is not related to skeletal muscle hypertrophy [39].

MSTN is a member of the transforming growth factor-β superfamily which suppresses satellite cell proliferation and differentiation [40] as well as MPS in mature muscle fibers [41]. Notably, skeletal muscle MSTN mRNA is down-regulated in response to acute resistance exercise [42, 43], and MSTN mRNA and protein levels are down-regulated in response to chronic resistance training [44, 45]. In the current study, serum MSTN levels decreased with training regardless of cluster, although mRNA levels remained unaltered. While the latter finding is difficult to reconcile with past literature, the decrease in serum MSTN levels continues to suggest that resistance training decreases facets of myostatin signaling.

An intriguing finding was PRE VL thickness being significantly lower in HI versus LOW responders, while POST VL thickness was greater in HI versus LOW responders. This finding could indicate that HI responders had not only a greater capacity for localized VL hypertrophy given lower pre-training muscle thickness, but a greater potential for muscle plasticity. Indeed, while increased ribosome biogenesis or reduced proteasome activity may be related to the latter, we also hypothesize that extracellular matrix components could be related to this phenomena. In this regard, others have demonstrated that cardiac muscle rapidly hypertrophies with pericardial removal [46], suggesting that connective tissue is highly influential in muscle tissue growth. Likewise, select rodent evidence suggests that gene expression profiles related to extracellular matrix remodeling are correlated with muscle fiber growth [47]. Hence, while these markers were not assayed herein, we speculate that an interesting future research direction would include interrogating if younger LOW responders possess features suggestive of a less malleable extracellular matrix (e.g., an increased expression of collagen-related genes or thicker connective tissue components at the micro- and macrostructure levels).

Finally, while there were clear individual responses regarding VL thickness changes, all clusters experiencing similar increases in lean body mass and lower body strength with training is a noteworthy finding. Alternatively stated, a broader theme from these data are that LOW individual responders can still experience positive training adaptations when a rigorous daily undulating periodization resistance training program is implemented.

### Experimental considerations

Experimental considerations should be noted herein. Notably, the original intent of this study was to examine the effects of L-leucine or different protein supplements on skeletal muscle hypertrophy versus a carbohydrate placebo. However, as reported in a prior investigation [17] and in Table 1, all supplemented groups experienced similar increases in type II fiber hypertrophy and the distribution of participants consuming various supplements was not different between clusters. Aside from this consideration, a critical methodological limitation was that acute or chronic MPS responses to training were not assessed. Although data exists suggesting the contrary [48], previous investigations have reported acute post-exercise signal transduction events associated with increased MPS (e.g., increased p706sk phosphorylation) or heightened MPS responses to an initial bout of resistance exercise predicts long-term hypertrophic responses [49, 50]. Hence, while speculative at best, we posit HI responders in the current study may have experienced greater a MPS response following each training session compared to LOW and MOD responders. Moreover, while our study examined PRE- and POST-intervention biopsy and food log data, individual hypertrophic responses are likely a result of various physiological, environmental, and psychosomatic factors that were not directly assessed and occur on a day-to-day basis (e.g., sleep habits, stress levels, etc.). Hence, future research implementing study designs that better address these topics are warranted. One unresolved limitation was that our POST biopsy was in relatively close proximity to the last training bout (72 hours), so this methodological constraint may have confounded certain findings in some participants (e.g., protein expression patterns, mRNA expression patterns, satellite cell activity). Finally, one interesting observation ripe for future exploration was noted herein in that lower baseline VL thickness in the HI versus LOW cluster was not reflected in lower respective type I and II fCSA values at baseline. As stated in the introduction, we posit this observation may be related to inter-individual differences in VL fiber number. For instance, if two subjects had 500,000 VL fibers but subject #1 had a VL thickness that was 20% greater than subject #2 at baseline then the muscle fibers in subject #1 would expected to be 20% larger. However, under this same VL thickness scenario, if subject #1 had 400,000 muscle fibers and individual #2 had 20% more fibers (480,000 muscle fibers) then both would be expected to possess similarly sized muscle fibers. Notably, this magnitude of fiber difference between subjects is well within the realm of possibility according to Lexell’s data [19], and the potential inter-individual variation in fiber number illustrates why hypertrophic imaging analysis (e.g., ultrasound data) may not agree well with histological fCSA data, and future studies should attempt to address this issue.

## Conclusions

These data continue to describe factors which are associated with the individual hypertrophic responses to resistance training. Individuals with lower pre-training VL thickness values and greater increases muscle total RNA levels following 12 weeks of resistance training experienced greater VL muscle growth, although these biomarkers individually explained only ~8–11% of the variance in hypertrophy. We contend that research efforts continuing to identify significant predictors of hypertrophy will enable determination of whether such variables can be modulated on an individual basis in order to optimize exercise adaptations.

## Supporting information

S1 Fig Representative histology and western blot images. (TIF)

S1 File Analytical methods. (DOCX)

S2 File Raw data. (XLSX)
