# Supplementary figures and images for: Biomarkers associated with low, moderate, and high vastus lateralis muscle hypertrophy following 12 weeks of resistance training
Source: PLoS One. 2018 Apr 5;13(4):e0195203. doi: 10.1371/journal.pone.0195203 (PMC5886420; doi:10.1371/journal.pone.0195203)

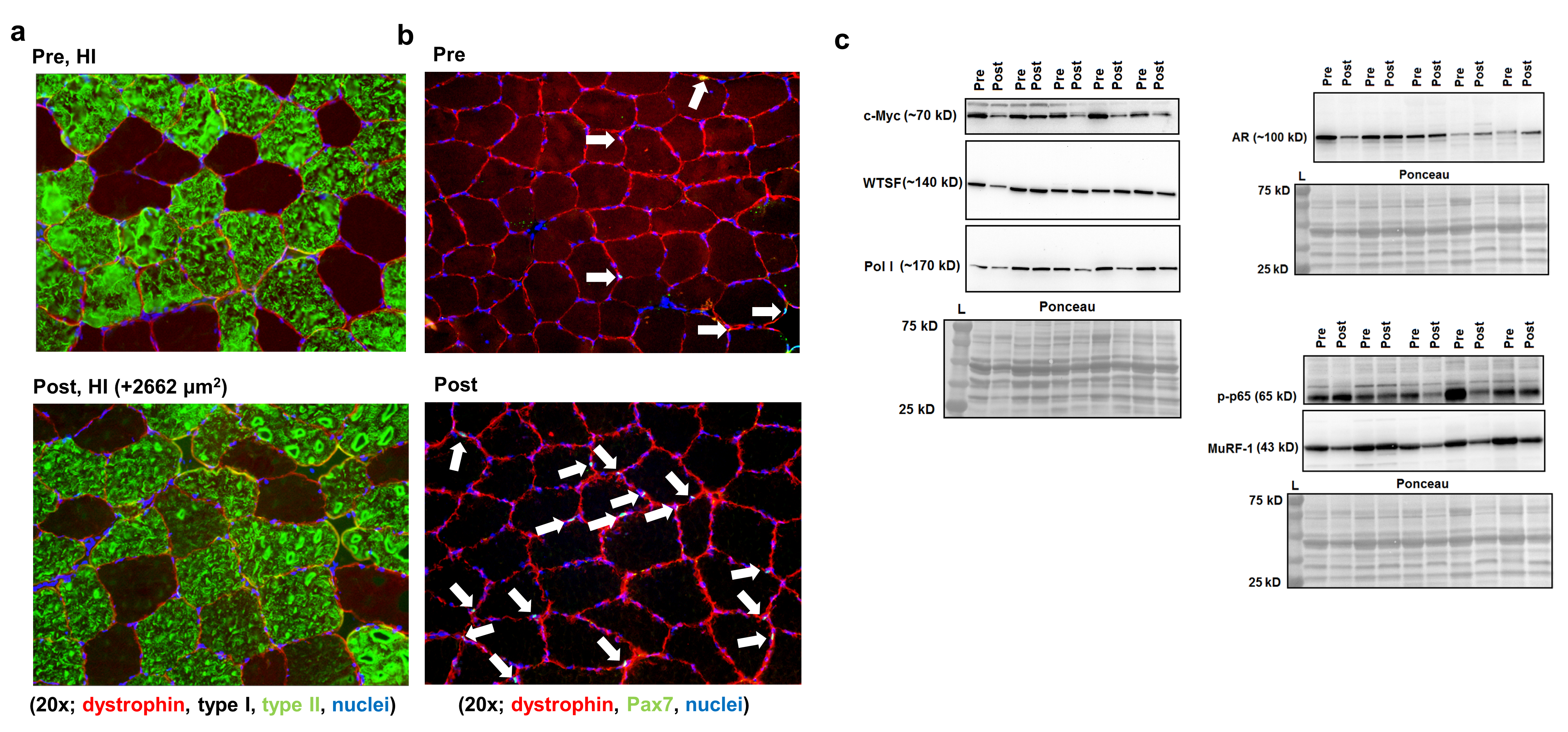

Supplement: S1 Fig — (TIF) [file pone.0195203.s001.tif]
