# Supplementary material for: Biomarkers associated with low, moderate, and high vastus lateralis muscle hypertrophy following 12 weeks of resistance training
Source: PLoS One. 2018 Apr 5;13(4):e0195203. doi: 10.1371/journal.pone.0195203 (PMC5886420; doi:10.1371/journal.pone.0195203)
Supplement: S1 File — (DOCX) [file pone.0195203.s002.docx]

**Supplemental Methods**

**Body composition testing**

During the PRE and POST testing sessions participants were instructed to submit a urine sample (~5 mL) to assess normal hydration specific gravity levels (1.005-1.020 ppm) using a handheld refractometer (ATAGO; Bellevue, WA, USA). Participants with a urine specific gravity >1.020 were asked to consume tap water every 15 min for 30 min and then were re-tested. Following hydration testing, height and body mass were assessed using a digital column scale (Seca 769; Hanover, MD, USA) with weights and heights collected to the nearest 0.1 kg and 0.5 cm, respectively. Next, participants were subjected to a full body DXA scan (Lunar Prodigy; GE Corporation, Fairfield, Connecticut, USA) while wearing general sports attire (i.e., athletic shorts or compression shorts and an athletic shirt) to assess various body composition characteristics. According to previous data published by our laboratory, the same-day reliability of the DXA during a test-calibrate-retest on 10 participants produced an intra-class correlation coefficient of 0.998 for total body lean mass (mean±SE difference between tests = 0.29±0.13 kg) [1].

Following DXA scans, participants were subjected to an ultrasound assessment to determine VL muscle thickness with a 3 to 12 MHz multi-frequency linear phase array transducer (Logiq S7 R2 Expert; General Electric, Fairfield, CT USA). Measurements were obtained from the midway point between the iliac crest and patella of the right femur whereby participants were in a standing position and all weight was placed on the left leg. All DXA scans and ultrasound assessments were completed by the same investigator (W.C.K., and P.W.M., respectively) as suggested by previous research interventions [2, 3] in order to minimize variability in testing procedures. The back-to-back day reliability VL thickness during a test-retest on 10 participants produced an intra-class correlation coefficient of 0.991 (mean±SE difference between tests = 0.037±0.023 cm).

**Tissue processing**

During PRE and POST muscle biopsy procurement, samples were processed immediately and stored for later analyses as described below. For histological analyses, approximately 20-40 mg from each biopsy specimen was embedded in cryomolds containing optimal cutting temperature (OCT) media (Tissue-Tek®, Sakura Finetek Inc; Torrence, CA, USA). Cryomolds were frozen using liquid nitrogen-cooled isopentane and stored at -80ºC until immunofluorescent staining for fCSA and myonuclear number described below. Embedding was performed per the methods of Kumar et al. [4] whereby tissue was laid in the cryomolds in the correct orientation for perpendicular slicing in a non-stretched state prior to rapid freezing.

For protein analyses, approximately 50 mg from each biopsy specimen was placed in 1.7 mL microcentrifuge tubes containing 500 µL of ice-cold cell lysis buffer [20 mM Tris-HCl (pH 7.5), 150 mM NaCl, 1 mM Na_2_EDTA, 1 mM EGTA, 1% Triton; Cell Signaling, Danvers, MA, USA] pre-stocked with protease and Tyr/Ser/Thr phosphatase inhibitors (2.5 mM sodium pyrophosphate, 1 mM β-glycerophosphate, 1 mM Na_3_VO_4_, 1 µg/mL leupeptin). Samples were then homogenized by hand via micropestle manipulation, insoluble proteins were removed with centrifugation at 500 *g* for 5 minutes, and obtained sample lysates were stored at -80ºC prior to Western blotting (described below). While the lysis buffer did not contain harsh detergents in order to ensure complete nuclear lysis, the tight-fitting pestle method we implemented ensured that nuclear lysis occurred, and the presence of histone 2.Ax was confirmed in the lysates of a subset of lysates using Western blotting (1:1000; GeneTex, GTX108272, Irvine, CA USA).

For total RNA and real-time polymerase chain reaction (RT-PCR) analyses, approximately 20-30 mg from each biopsy specimen was weighed using an analytical scale with a sensitivity of 0.0001 g (Mettler-Toledo; Columbus, OH, USA). Tissue was then homogenized in 1.7 mL microcentrifuge tubes containing 500 µL of Ribozol (Ameresco; Solon, OH, USA) via micropestle manipulation and RNA isolation was performed per manufacturer recommendations. Samples were then frozen at -80ºC until RNA quantification and cDNA synthesis (described below). Total RNA per unit muscle weight was used as a surrogate for ribosome density as in past publications [5-7], and changes in total RNA were presumed to represent changes in ribosome density herein.

**Immunofluorescent histochemistry for type II fCSA and satellite cell count determination**

Methods for immunofluorescent histochemistry have been employed previously in our laboratory and described elsewhere [8-10]. Briefly, sections from OCT‐preserved samples were cut at a thickness of 20 μm using a cryotome (Leica Biosystems; Buffalo Grove, IL, USA) and were adhered to positively-charged histology slides. Once all samples were sectioned, batch processing occurred for immunofluorescent histochemistry. During batch processing sections were air-dried at room temperature for 30 minutes, fixed with 10% formalin for 10 minutes, permeabilized in a phosphate‐buffered saline (PBS) solution containing 0.5% Triton X‐100, and blocked with 100% Pierce Super Blocker (Thermo Fisher Scientific) for 25 minutes.

For fiber type staining, sections were subsequently washed for 5 minutes in PBS. Sections were then incubated for 1 hour with a pre-diluted commercially-available rabbit primary anti-dystrophin IgG antibody solution (Thermo Fisher Scientific) and spiked in mouse anti-myosin II IgG (catalog #: SC71 supernatant; Hybridoma Bank, Iowa City, IA, USA; 100 µL added per 1 mL of dystrophin antibody solution). Sections were then washed for 5 minutes in PBS and incubated in the dark for 1 hour with a secondary antibody solution containing Texas Red-conjugated anti-rabbit IgG (Vector Laboratories, Burlingame, CA, USA), and Alexa Fluor 488-conjugated anti-mouse IgG (Thermo Fisher Scientific) (10 µL of all secondary antibodies per 1 mL of blocking solution). Sections were washed for 5 minutes in PBS, air-dried and mounted with fluorescent media containing 4,6-diamidino-2-phenylindole (DAPI; Vector Laboratories). Following mounting, slides were stored in the dark at 4ºC until immunofluorescent images were obtained.

For satellite cell staining, separate sections were incubated for 1 hour with a pre-diluted commercially-available rabbit primary anti-dystrophin IgG antibody solution (Thermo Fisher Scientific) and spiked-in mouse anti-Pax7 IgG (catalog #: PAX7 supernatant; Hybridoma Bank; 67 µL added per 1 mL of dystrophin antibody solution). Sections were then washed for 5 minutes in 1x PBS and incubated in the dark for 1 hour with a secondary antibody solution containing Texas Red-conjugated anti-rabbit IgG (Vector Laboratories), and Alexa Fluor 488-conjugated anti-mouse IgG (Thermo Fisher Scientific) (10 µL of all secondary antibodies per 1 mL of blocking solution). Sections were then washed for 5 minutes in PBS, air-dried, and were mounted with fluorescent media containing DAPI (Vector Laboratories). Following mounting, slides were stored in the dark at 4ºC until immunofluorescent images were obtained.

After staining was performed on all sections, digital images were captured using a fluorescence microscope (Nikon Instruments, Melville, NY, USA) and 20x objective. Approximate exposure times were 600 ms for red and green imaging and 30 ms for blue imaging. For fiber typing, our staining method allowed the identification of cell membranes (detected by the Texas Red filter), type II fiber green cell bodies (detected by the FITC filter), type I fiber black cell bodies (unlabeled), and myonuclei (detected by the DAPI filter). For satellite cell identification our staining method allowed the identification of cell membranes (detected by the Texas Red filter), small green cell bodies as satellite cells (detected by the FITC filter), and myonuclei (detected by the DAPI filter). Measurements of type I and II fCSA were performed using the open-sourced software CellProfiler^TM^ [11] per modified methods previously described whereby the number of pixels counted within the border of each muscle fiber were converted to a total area (µm^2^) [8]. Measurements of fiber type-specific myonuclear number were also performed using open-sourced software CellProfiler^TM^ which was able to discriminate the fiber border that corresponded to each myonuclei [11]. Satellite cells were manually counted using a grid function in the NIS Elements software (Nikon Instruments) and handheld tally counter. Per the recommendations of Mackey et al. [12], at least 50 fibers per specimen were quantified in order to obtain accurate fCSA, myonuclear number and satellite cell values. Representative 20x micrographs for fiber type-specific CSA and satellite cell analyses are found in supporting information (panels a&b in S1 Fig. Representative histology and Western blot images).

**Western blotting**

Whole-tissue sample lysates obtained through cell lysis buffer processing (described above) were batch process-assayed for total protein content using a BCA Protein Assay Kit (Thermo Fisher Scientific). Lysates were then prepared for Western blotting using 4x Laemmli buffer at 1 µg/µL. Following sample preparation, 25 µL samples were loaded onto 4-15% SDS-polyacrylamide gels (Bio-Rad, Hercules, CA, USA) and subjected to electrophoresis (180 V for 45-60 minutes) using pre-made 1x SDS-PAGE running buffer (Ameresco). Proteins were then transferred (200 mA for 2 hours) to polyvinylidene difluoride membranes (Bio-Rad), Ponceau stained and imaged to ensure equal protein loading between lanes. Membranes were then blocked for 1 hour at room temperature with 5% nonfat milk powder in Tris-buffered saline with 0.1% Tween-20 (TBST; Ameresco). Rabbit anti-human Pol I (1:1000; Thermo Fisher Scientific, PA5-39139), mouse anti-human UBF (1:500; Santa Cruz Biotechnology, sc-13125, Dallas, TX, USA), rabbit anti-human c-Myc (1:1000; Cell Signaling, #9402), rabbit anti-human WSTF (1:1000; Cell Signaling, #2152), rabbit anti-human phosphorylated p65/NF-κB (Ser536) (1:1000; Cell Signaling, #3031), rabbit anti-human androgen receptor (AR) (1:1000; Thermo Fisher Scientific, MA5-13426), and rabbit anti-human MuRF-1/Trim63 (1:2000; Abcam, ab172479, Danvers, MA, USA) were incubated with membranes overnight at 4º C in TBST with 5% bovine serum albumin (BSA). The following day, membranes were incubated with horseradish peroxidase-conjugated anti-rabbit or anti-mouse IgG (1:2000, Cell Signaling) in TBST with 5% BSA at room temperature for 1 hour. Membrane development was performed using an enhanced chemiluminescent reagent (Luminata Forte HRP substrate; EMD Millipore, Billerica, MA, USA), and band densitometry was performed using a gel documentation system and associated densitometry software (UVP, Upland, CA, USA). Densitometry values for all protein targets were normalized to Ponceau densities. All values for a given protein target were normalized to the LOW PRE group mean values whereby the LOW PRE group average was 1.00, and data were expressed as relative expression units (REU’s). A representative membrane from all blots are presented in supporting information (panel c in S1 Fig. Representative histology and Western blot images).

**RNA concentration determination and real-time PCR**

Total RNA concentrations were determined in duplicate from isolated RNA (described above) using a NanoDrop Lite spectrophotometer (Thermo Fisher Scientific). 1 µg of RNA was reverse transcribed into cDNA for RT-PCR analysis with cDNA synthesis reagents (Quanta Biosciences, Gaithersburg, MD) per the manufacturer’s recommendations. RT-PCR was performed using gene-specific primers and SYBR green chemistry (Quanta Biosciences). Primer sequences used were as follows: 45S pre-rRNA forward primer 5’- GAACGGTGGTGTGTCGTT-3’, reverse primer 5’-GCGTCTCGTCTCGTCTCACT-3’; tumor necrosis factor-alpha (TNF-α): forward primer 5’-tccttcagacaccctcaacc-3’, reverse primer 5’-aggccccagtttgaattctt-3’; interleukin-1beta (IL-1β): forward primer 5’-aaggcggccaggatataact-3’, reverse primer 5’-ccctagggattgagtccaca-3’; IL-6: forward primer 5’-aggagacttgcctggtgaaa-3’, reverse primer 5’-caggggtggttattgcatct-3’; myostatin (MSTN): forward primer 5’- GACCAGGAGAAGATGGGCTGAATCCGTT-3’, reverse primer 5’- CTCATCACAGTCAAGACCAAAATCCCTT-3’; fibrillarin (FBL, housekeeping gene 1): forward primer 5’- CCCACACCTTCCTGCGTAAT-3’ reverse primer 5’-GCTGAGGCTGTGGAGTCAAT-3’; cyclophilin (PPIA, housekeeping gene 2): forward primer 5’-CGATGTCTCAGAGCACGAAA-3’, reverse primer 5’-CCCACCTGTTTCTTCGACAT-3’. 2^−ΔCq^ values for each gene of interest were calculated whereby ΔCq = gene of interest Cq – (geometric mean of FBL and PPIA Cq). All values for a given mRNA target were normalized to the LOW PRE group mean values whereby the LOW PRE group average was 1.00, and data were expressed as REU’s. Prior melt curve analyses from our laboratory confirmed that only one RT-PCR product was obtained with the primer sets being used. Select participants from each cluster were removed from analyses if PRE to POST geometric mean of FBL and PPIA Cq values deviated by ±2.00 Cq. Removal of these participants ensured that PRE and POST geometric mean of FBL and PPIA Cq values did not differ between clusters or change over time. The final n-sizes for mRNA analyses were n=12 LOW, n=25 MOD, and n=18 HI.

**20S proteasome activity assays**

20S proteasome activity assays on sample supernatants were performed using commercially available fluorometric kits (BioVision Inc., Milpitas, CA, USA) per the manufacturer’s instructions which are similar to methods previously published by our laboratory [13]. All fluorometric readings were all normalized to the LOW PRE group mean values whereby the LOW PRE group average was 1.00, and data were expressed as relative activity. The average coefficient of variation values for all duplicates were ~2%. Due to the lack of an adequate volume of cell lysates for all samples, partial n-sizes were analyzed per cluster (n-sizes indicated in figures).

**Serum assays**

Serum total testosterone and cortisol were analyzed using commercially available kits (ALPCO, Salem, NH, USA). Serum MSTN was analyzed using commercially available kits (R&D Systems, Minneapolis, MN, USA). Coefficient of variation values for all duplicates were 4.6% for total testosterone, 3.4% for cortisol, and 3.4% for MSTN. Due to resource constraints, partial n-sizes were analyzed per cluster for each target n-sizes indicated in figures). Notably, serum IL-6 assays were attempted using commercially available kits (ALPCO), but all readings were below the sensitivity of the assay and were not reported.

**References**

1. Kephart WC, Wachs TD, Mac Thompson R, Brooks Mobley C, Fox CD, McDonald JR, et al. Ten weeks of branched-chain amino acid supplementation improves select performance and immunological variables in trained cyclists. Amino Acids. 2016;48(3):779-89. doi: 10.1007/s00726-015-2125-8. PubMed PMID: 26553453.

2. Lohman M, Tallroth K, Kettunen JA, Marttinen MT. Reproducibility of dual-energy x-ray absorptiometry total and regional body composition measurements using different scanning positions and definitions of regions. Metabolism. 2009;58(11):1663-8. doi: 10.1016/j.metabol.2009.05.023. PubMed PMID: 19632696.

3. Lockwood CM, Roberts MD, Dalbo VJ, Smith-Ryan AE, Kendall KL, Moon JR, et al. Effects of Hydrolyzed Whey versus Other Whey Protein Supplements on the Physiological Response to 8 Weeks of Resistance Exercise in College-Aged Males. J Am Coll Nutr. 2017;36(1):16-27. doi: 10.1080/07315724.2016.1140094. PubMed PMID: 27710436.

4. Kumar A, Accorsi A, Rhee Y, Girgenrath M. Do's and don'ts in the preparation of muscle cryosections for histological analysis. J Vis Exp. 2015;(99):e52793. doi: 10.3791/52793. PubMed PMID: 26066009; PubMed Central PMCID: PMCPMC4542757.

5. Nader GA, McLoughlin TJ, Esser KA. mTOR function in skeletal muscle hypertrophy: increased ribosomal RNA via cell cycle regulators. Am J Physiol Cell Physiol. 2005;289(6):C1457-65. doi: 10.1152/ajpcell.00165.2005. PubMed PMID: 16079186.

6. Mobley CB, Mumford PW, Kephart WC, Conover CF, Beggs LA, Balaez A, et al. Effects of testosterone treatment on markers of skeletal muscle ribosome biogenesis. Andrologia. 2016;48(9):967-77. doi: 10.1111/and.12539. PubMed PMID: 26781353.

7. Mobley CB, Fox CD, Thompson RM, Healy JC, Santucci V, Kephart WC, et al. Comparative effects of whey protein versus L-leucine on skeletal muscle protein synthesis and markers of ribosome biogenesis following resistance exercise. Amino Acids. 2016;48(3):733-50. doi: 10.1007/s00726-015-2121-z. PubMed PMID: 26507545.

8. Mobley CB, Haun CT, Roberson PA, Mumford PW, Romero MA, Kephart WC, et al. Effects of Whey, Soy or Leucine Supplementation with 12 Weeks of Resistance Training on Strength, Body Composition, and Skeletal Muscle and Adipose Tissue Histological Attributes in College-Aged Males. Nutrients. 2017;9(9). doi: 10.3390/nu9090972. PubMed PMID: 28869573.

9. Martin JS, Kephart WC, Haun CT, McCloskey AE, Shake JJ, Mobley CB, et al. Impact of external pneumatic compression target inflation pressure on transcriptome-wide RNA expression in skeletal muscle. Physiol Rep. 2016;4(22). doi: 10.14814/phy2.13029. PubMed PMID: 27884954; PubMed Central PMCID: PMCPMC5357997.

10. Hyatt HW, Toedebusch RG, Ruegsegger G, Mobley CB, Fox CD, McGinnis GR, et al. Comparative adaptations in oxidative and glycolytic muscle fibers in a low voluntary wheel running rat model performing three levels of physical activity. Physiol Rep. 2015;3(11). doi: 10.14814/phy2.12619. PubMed PMID: 26603455; PubMed Central PMCID: PMCPMC4673647.

11. Carpenter AE, Jones TR, Lamprecht MR, Clarke C, Kang IH, Friman O, et al. CellProfiler: image analysis software for identifying and quantifying cell phenotypes. Genome Biol. 2006;7(10):R100. doi: 10.1186/gb-2006-7-10-r100. PubMed PMID: 17076895; PubMed Central PMCID: PMCPMC1794559.

12. Mackey AL, Kjaer M, Charifi N, Henriksson J, Bojsen-Moller J, Holm L, et al. Assessment of satellite cell number and activity status in human skeletal muscle biopsies. Muscle Nerve. 2009;40(3):455-65. doi: 10.1002/mus.21369. PubMed PMID: 19705426.

13. Mobley CB, Mumford PW, Kephart WC, Haun CT, Holland AM, Beck DT, et al. Aging in Rats Differentially Affects Markers of Transcriptional and Translational Capacity in Soleus and Plantaris Muscle. Front Physiol. 2017;8:518. doi: 10.3389/fphys.2017.00518. PubMed PMID: 28775694; PubMed Central PMCID: PMCPMC5517446.
